# Author Correction: Microbially induced potassium enrichment in Paleoproterozoic shales and implications for reverse weathering on early Earth

**DOI:** 10.1038/s41467-019-11042-x

**Published:** 2019-06-27

**Authors:** Jérémie Aubineau, Abderrazak El Albani, Andrey Bekker, Andrea Somogyi, Olabode M. Bankole, Roberto Macchiarelli, Alain Meunier, Armelle Riboulleau, Jean-Yves Reynaud, Kurt O. Konhauser

**Affiliations:** 1 UMR 7285 CNRS IC2MP, University of Poitiers, Poitiers, 86073 France; 2 Department of Earth and Planetary Sciences, University of California, Riverside, CA 92521 USA; 3 Nanoscopium Beamline Synchrotron Soleil, BP 48, Saint-Aubin, Gif-sur-Yvette, 91192 France; 4 Department of Geosciences, University of Poitiers, Poitiers, 86073 France; 5 Department of Prehistory, UMR 7194 CNRS, National Museum of Natural History, Paris, 75005 France; 6 UMR 8187 CNRS LOG, University of Lille, ULCO, Villeneuve d’Ascq, 59655 France; 7 Department of Earth and Atmospheric Sciences, University of Alberta, Edmonton, AB T6G 2E3 Canada

**Keywords:** Geochemistry, Palaeontology, Sedimentology

Correction to: *Nature Communications* 10.1038/s41467-019-10620-3, published online 17 June 2019.

The original version of this Article contained an error in the spelling of the author Abderrazak El Albani, which was incorrectly given as Abderrazak El Abani. This has now been corrected in both the PDF and HTML versions of the Article.

