# Supplementary material for: Microbially induced potassium enrichment in Paleoproterozoic shales and implications for reverse weathering on early Earth
Source: Nat Commun. 2019 Jun 17;10:2670. doi: 10.1038/s41467-019-10620-3 (PMC6572813; doi:10.1038/s41467-019-10620-3)
Supplement: Supplementary file 1 — Supplementary Information [file 41467_2019_10620_MOESM1_ESM.pdf]

Supplementary Information

Microbially induced potassium enrichment in Paleoproterozoic shales and implications for reverse weathering on early Earth

Authors:

Jérémie Aubineau et al.

Supplementary Note 1

Geochemical comparisons of selected major elements between MRS and host sediments.

Al_2O_3 content reveals a negative relationship with SiO_2 in terms of clastic grain size (Supplementary Fig. 4a), with shales being more aluminous than sandstone. This is to be expected, but surprisingly, non-pyritized MRS also have a higher SiO_2 content and K_2O/SiO_2 ratio than pyritized MRS (Fig. 3), indicating that quartz dilution is unlikely. Moreover, the Francevillian FB_2 Member shales do not contain silica excess with respect to the average shale (Supplementary Table 1)[1], inconsistent with extensive silicification during early diagenesis. Al_2O_3 displays a positive correlation with K_2O in pyritized MRS and host sediments, but shows no trend for non-pyritized MRS (Supplementary Fig. 4b), suggesting the presence of K-poor clays. Lithologies show different K_2O and Na_2O relationships, but, on the average, K_2O and Na_2O contents co-vary (Supplementary Fig. 4c), consistent with a similar relationship shown by chemical analyses of fine-grained illite[2]. MgO exhibits no significant correlation with Al_2O_3 (Supplementary Fig. 4d), indicating the presence of Mg-bearing carbonate and/or Mg-poor phyllosilicates. Collectively, these binary plots highlight that the variations in major element content are controlled by mineralogy (*e.g.*, type of detrital and/or authigenic clays present in lithologies).

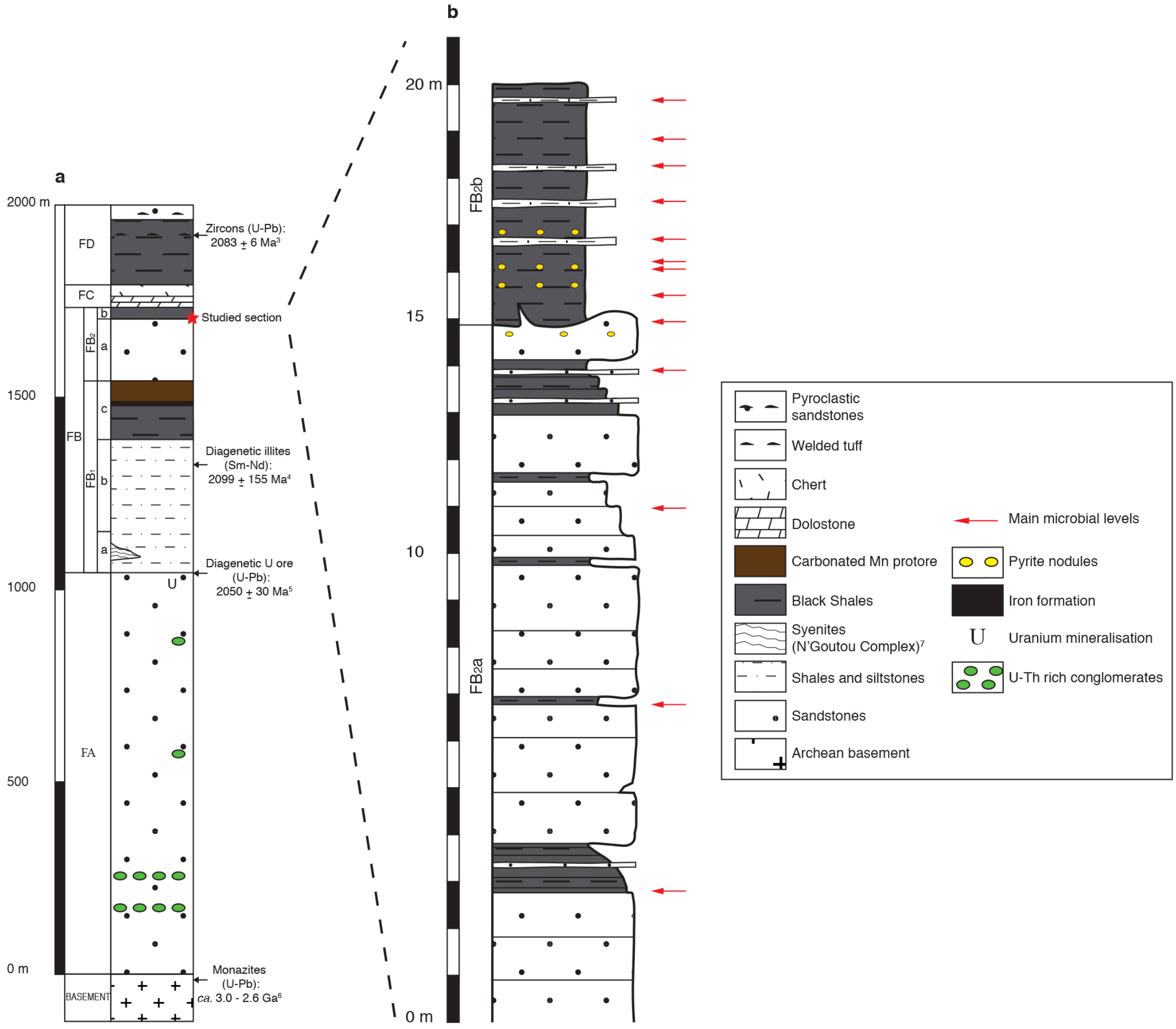

Supplementary Figure 1 | Lithostratigraphic columns for the Francevillian basin. a, Lithostratigraphy of the Paleoproterozoic Francevillian Series that comprises four sedimentary formations. **b**, Composite stratigraphic section of the FB_2 Member in the studied area. It consists of the 15 m thick coarse-grained sandstone conformably underlying the 5 m thick black shale sequence ([3]Horie *et al.*, 2005; [4]Bros *et al.*, 1992; [5]Gancarz, 1978; [6]Mouélé *et al.*, 2014; [7]Gauthier-Lafaye and Weber, 2003).

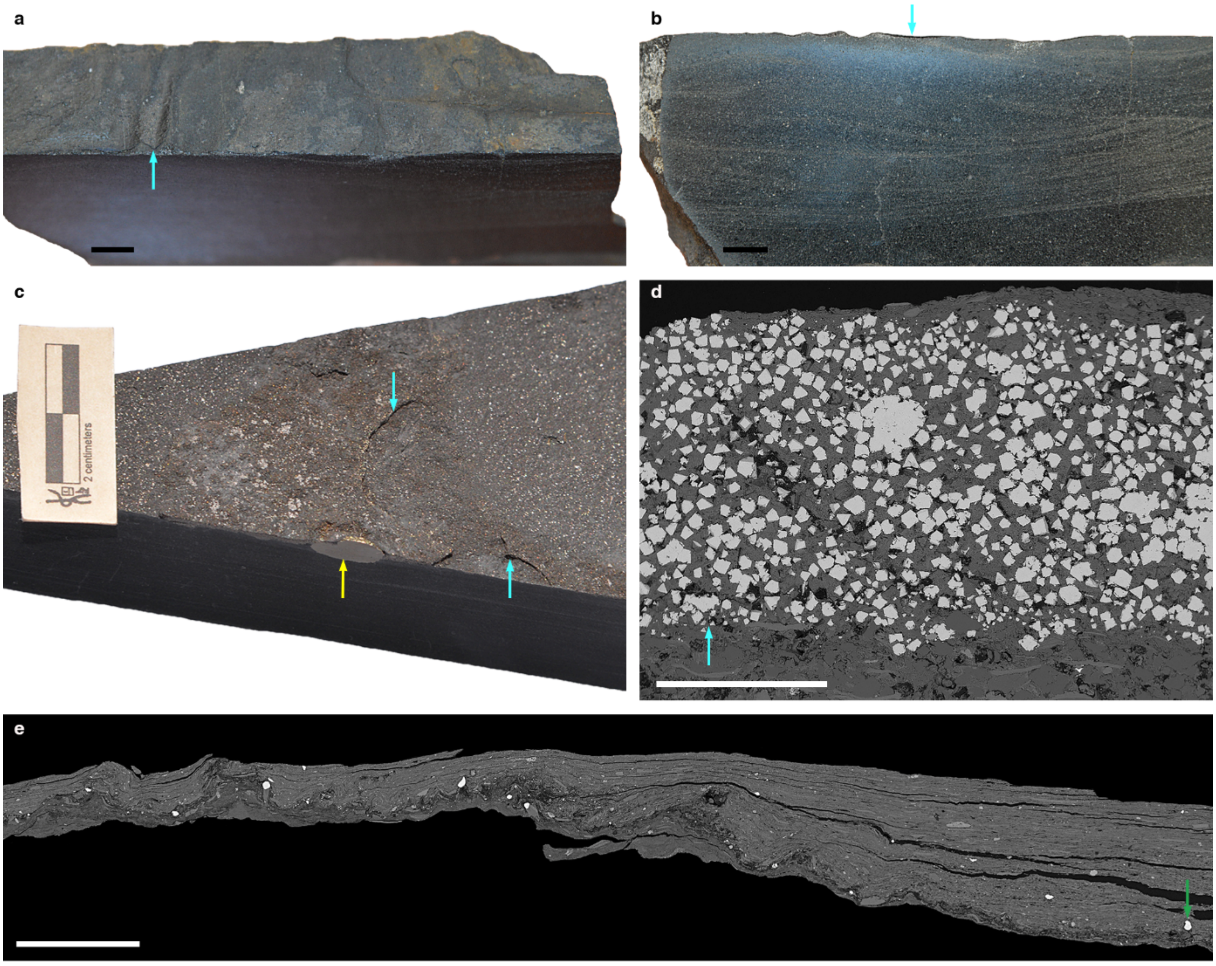

Supplementary Figure 2 | Microbial mat laminae and their host sediments. **a**, A µm-thick mat-related structure preserved as wrinkle on the top of the sandstone bed. **b**, Polished slab section of **a**. **c**, Pyritized microbial mat from the black shale facies. **d**, SEM imaging of **c**. Note extensively developed euhedral pyrite, making difficult to separate black shale from pyritized mat structures. **e**, SEM image of a thinly laminated mat from the top of the sandstone bed. Note the microtexture of mat laminae. Blue, yellow, and green arrows point to the thin biofilm layers, macrofossil[8,9], and heavy minerals, respectively. Scale bars are 1 cm (**a**, **b**), and 0.5 mm (**d**, **e**).

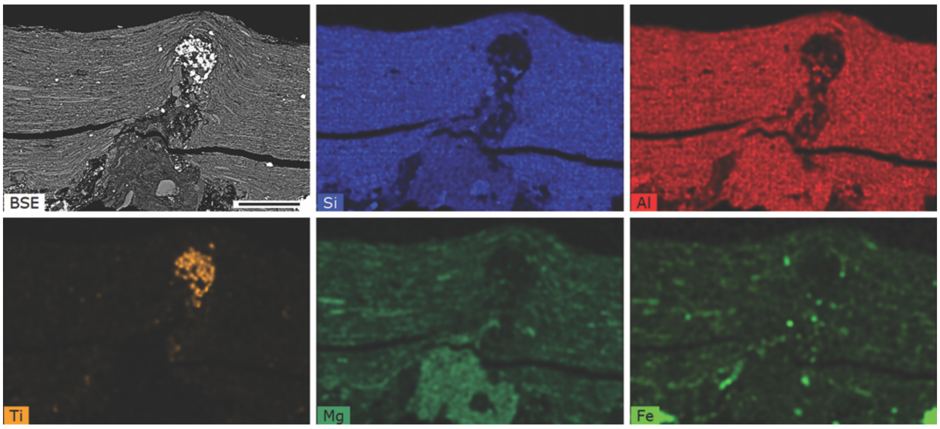

Supplementary Figure 3 | Petrography, SEM, and EDX of bubble-like features in cross-section perpendicular to the bedding plane. Back-scattered electron (BSE) and composite (Si, Al, Ti, Mg, and Fe) elemental maps show mineral composition of circular structures within the MRS. The cone-like structure mainly consists of Al- and Mg-rich clays. Scale bar is 200 µm.

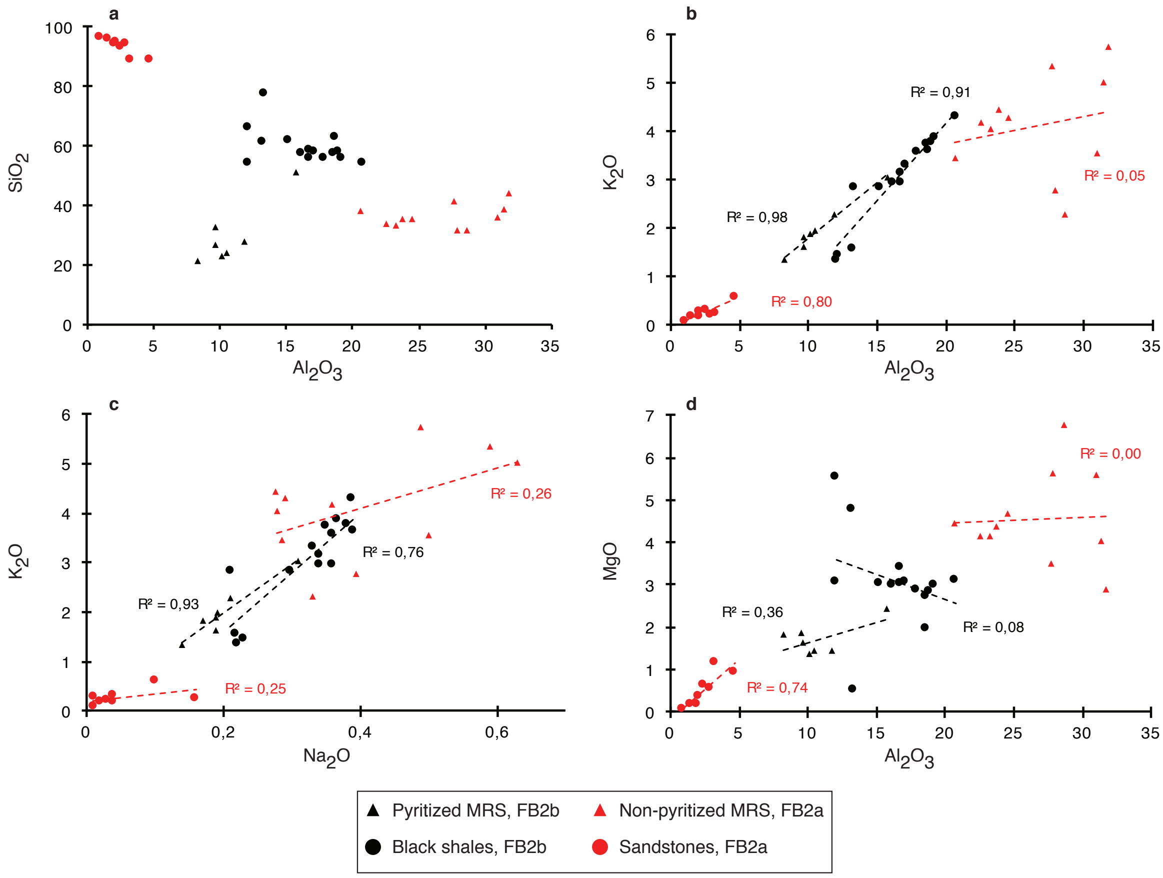

Supplementary Figure 4 | Cross plots of selected major elements. a, SiO_2 vs. Al_2O_3. **b**, K_2O vs. Al_2O_3. **c**, K_2O vs. Na_2O. **d**, MgO vs. Al_2O_3.

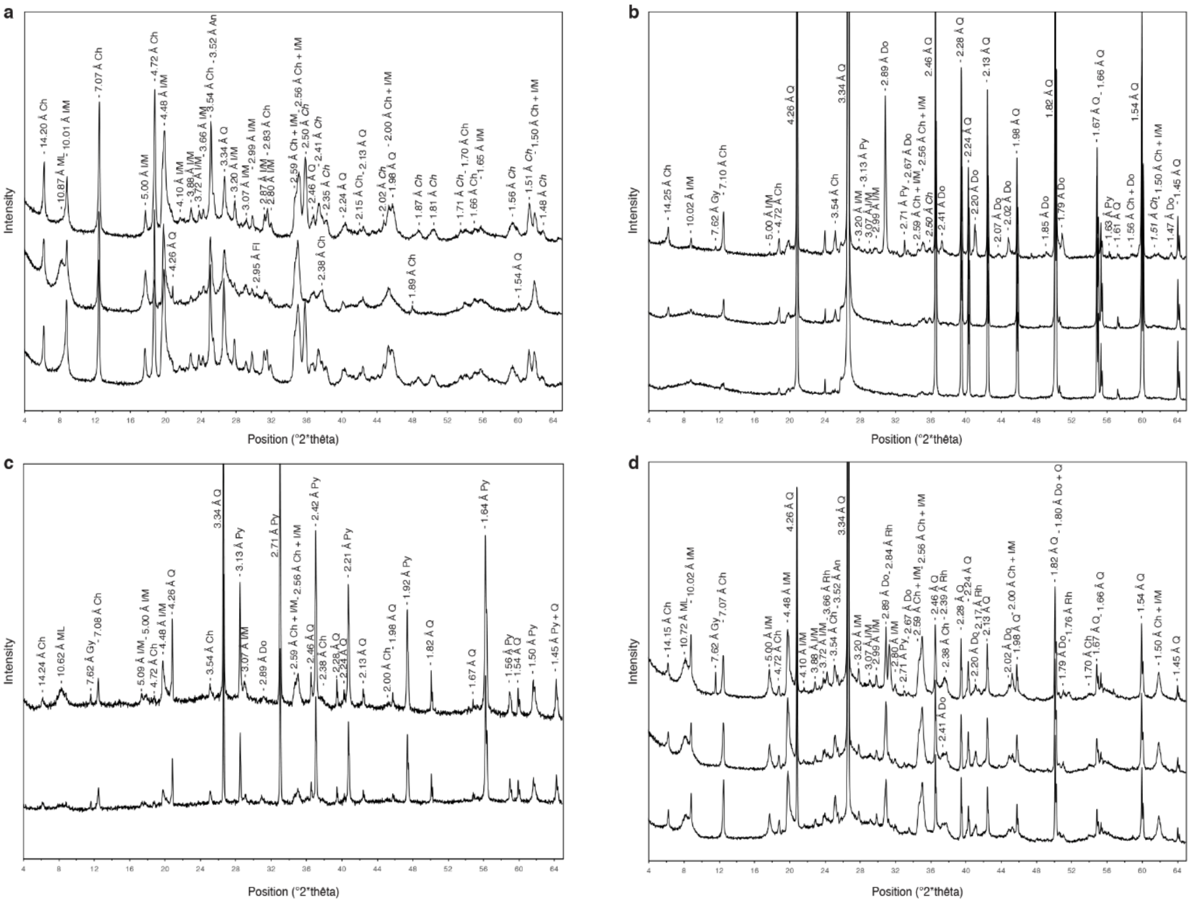

Supplementary Figure 5 | XRD patterns of representative randomly oriented powders. a, Non-pyritized microbial mats. **b**, Sandstones. **c**, Pyritized biofilms. **d**, Black shales. [Chlorite (Ch); Mixed-layer (ML); Illite/mica (I/M); Quartz (Q); Anatase (An); Florencite (Fl); Di-tri-octahedral chlorite[10] (*Ch*); Gypsum (Gy); Pyrite (Py); Dolomite (Do); Rhodochrosite (Rh)].

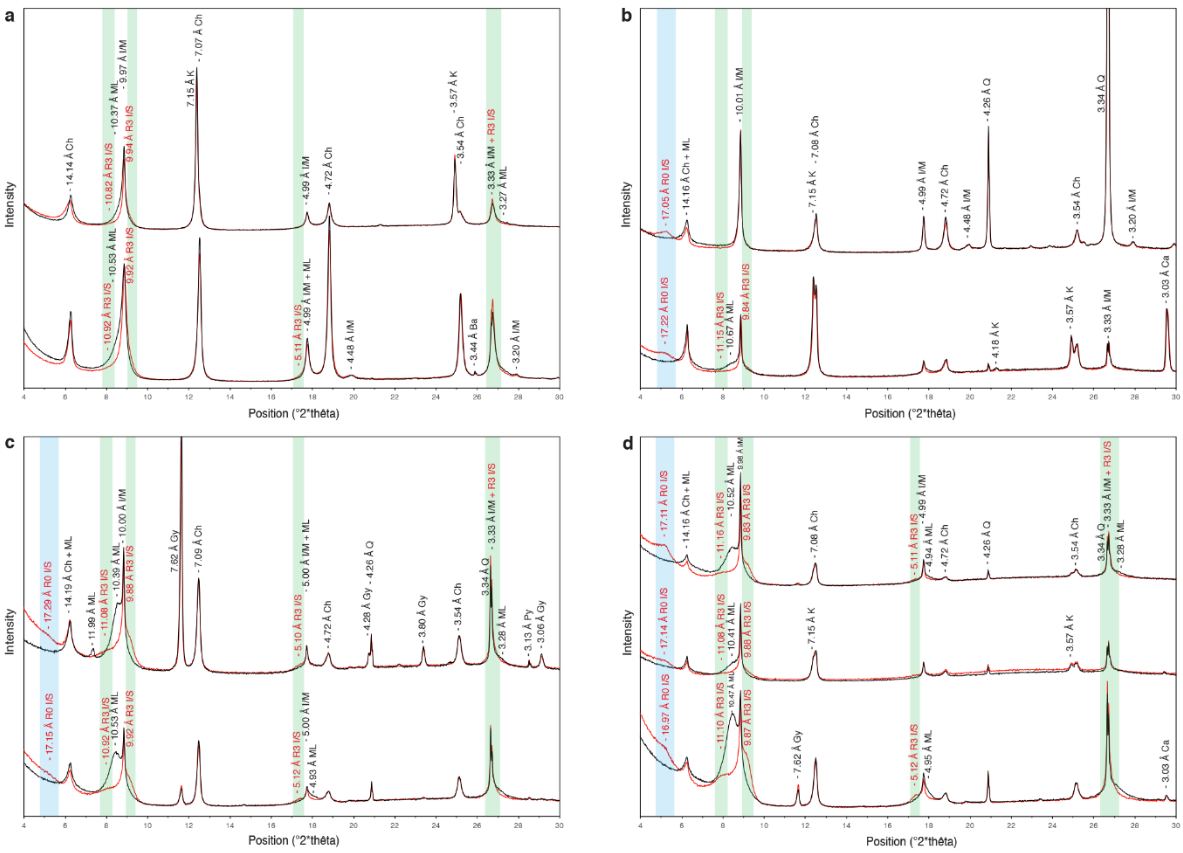

Supplementary Figure 6 | XRD patterns of representative oriented <2 µm clay fraction after air-dried treatment (black lines) and glycolation (red lines). **a**, Non-pyritized microbial mats. **b**, Sandstones. **c**, Pyritized biofilms. The weak signature of short-range ordered I-S MLMs likely indicates contamination from the underlying host sediment. **d**, Black shales. [Green areas correspond to long-range ordered (R3) I-S MLMs; Blue areas represent the randomly ordered (R0) I-S MLMs; Chlorite (Ch); Mixed-layer (ML); Illite/mica (I/M); Kaolinite (K); Quartz (Q); Barite (Ba); Calcite (Ca); Gypsum (Gy)].

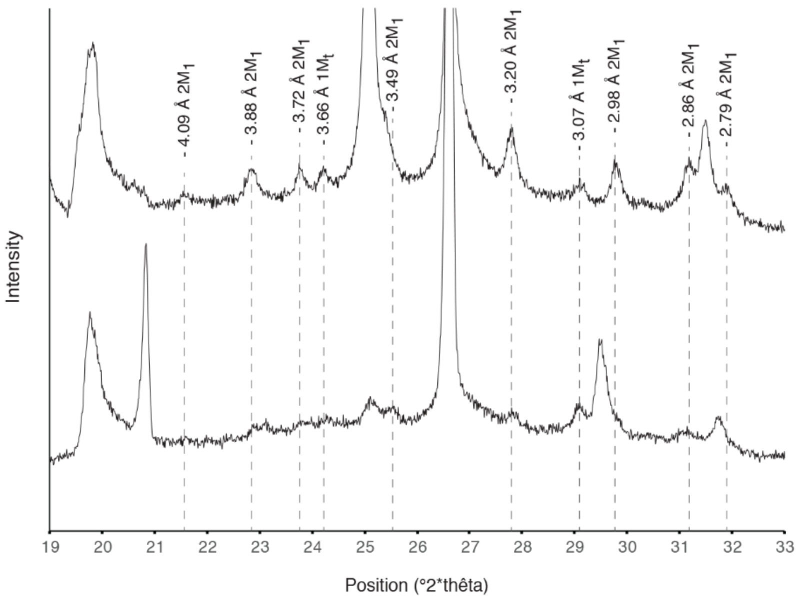

Supplementary Figure 7 | XRD patterns of randomly oriented powders (<2 μm clay fraction) for the MRS and host sediment are shown at the top and bottom, respectively. The *hkl* diffraction peaks are typical of illite polytypes with $2M_1$ and $1M_t$ polytypes, representing detrital and diagenetic illites, respectively.

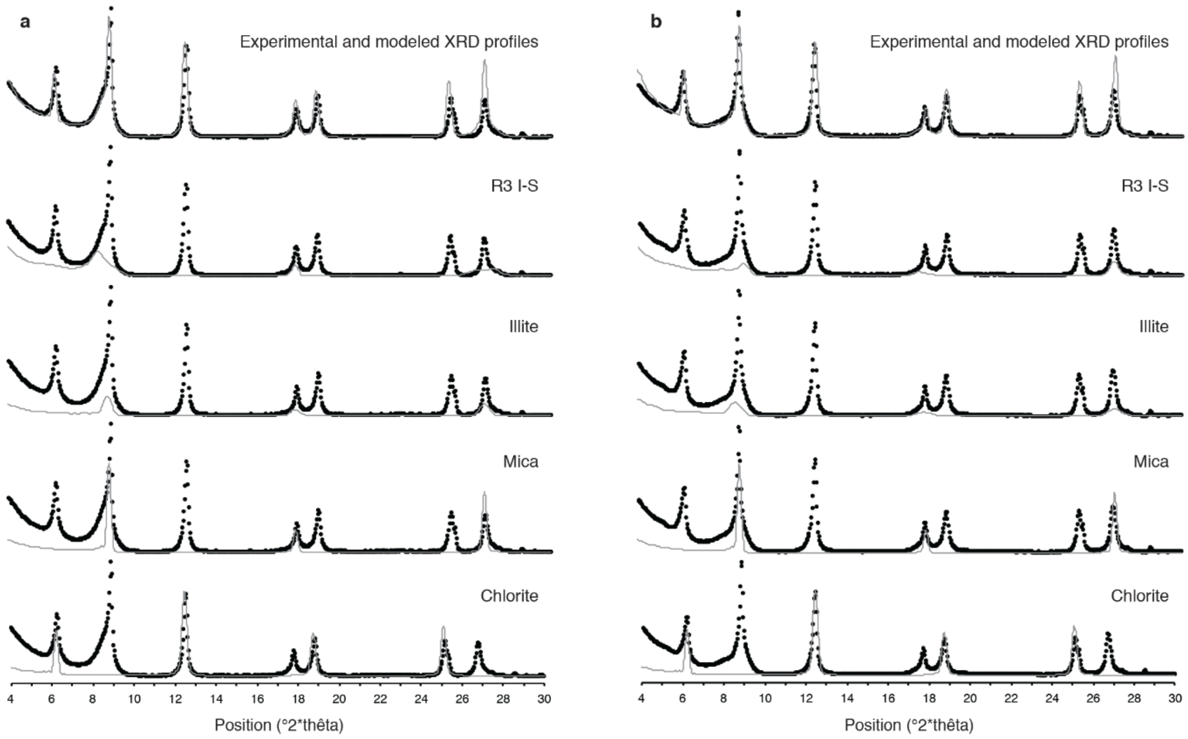

Supplementary Figure 8 | Experimental (crosses) and modeled (lines) XRD profiles of selected Ca-saturated MRS samples. Contribution of different minerals to the profiles is indicated. a, After air-dried preparation. **b**, After ethylene-glycol saturation.

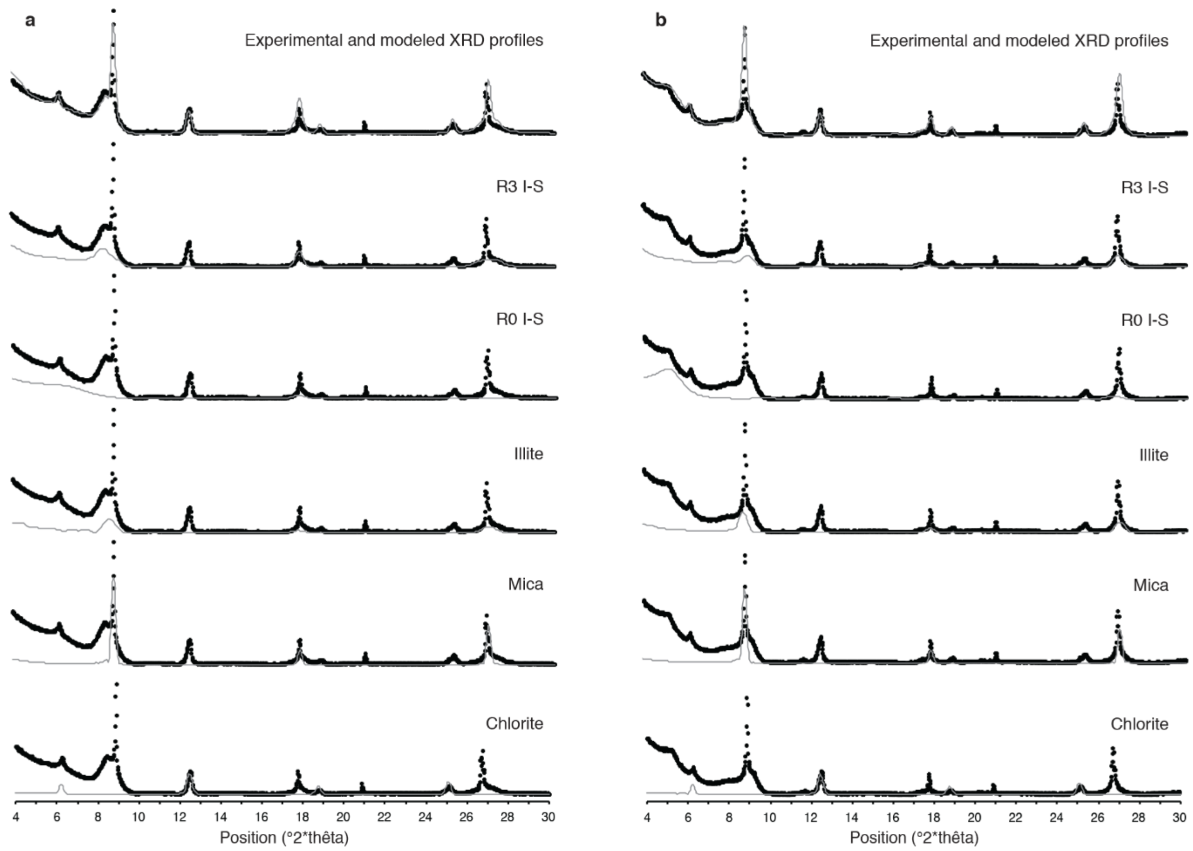

Supplementary Figure 9 | Experimental (crosses) and modeled (lines) XRD profiles of Ca-saturated host sediments. Contribution of different minerals to the profiles is indicated. a, After air-dried preparation. **b**, After ethylene-glycol saturation.

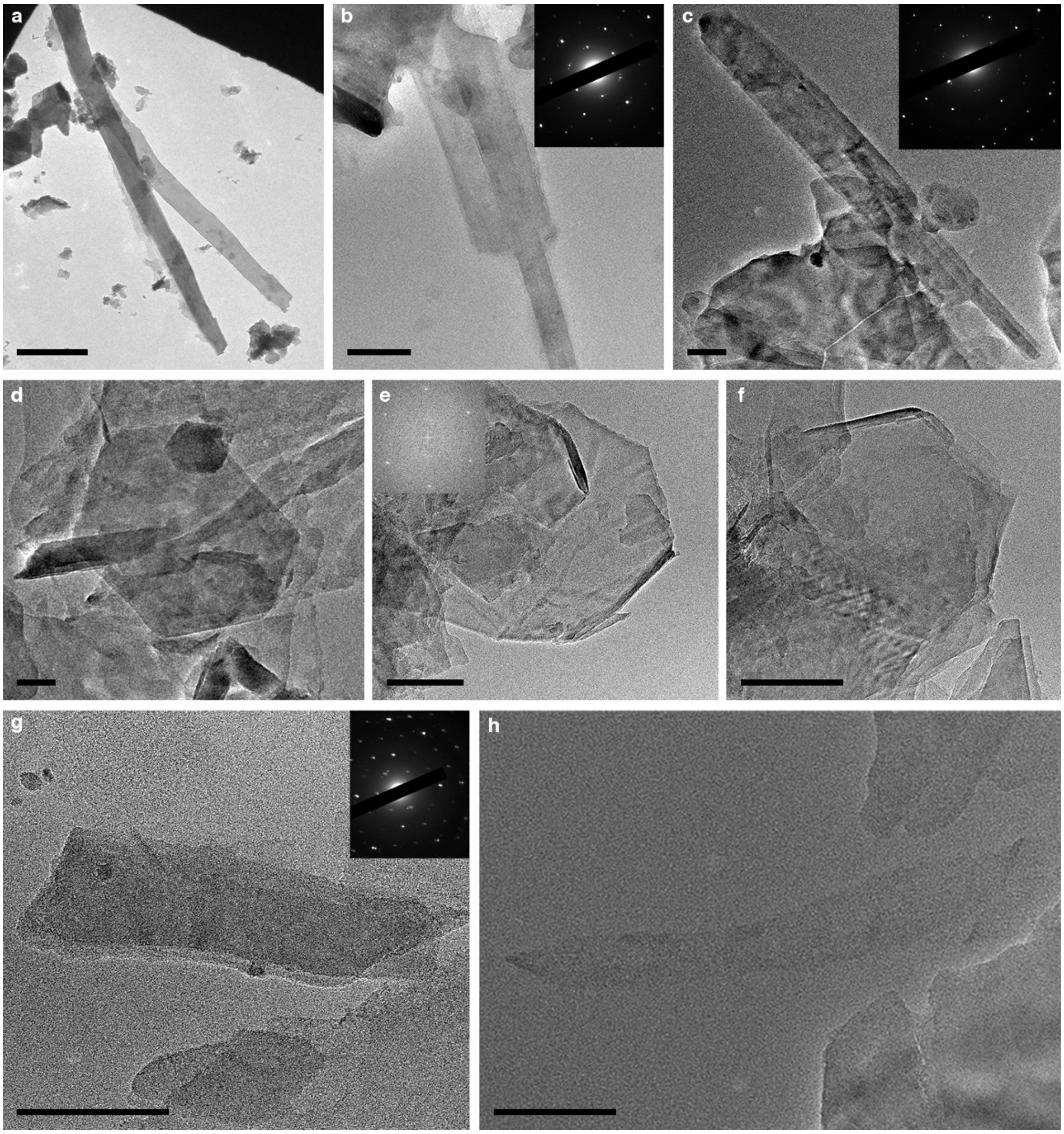

Supplementary Figure 10 | Transmission-electron images of illite minerals. a-c, Lath-shaped crystals from MRS. **d-f**, Hexagonal-shaped crystals observed in MRS. **g-h**, Lathlike and tiny lath-shaped crystals from host sediments. Scale bars are 2000 nm (**a**), 500 nm (**b**), 100 nm (**c**-**g**), 50 nm (**h**). The inset with Selected Area Electron Diffraction (SAED) pattern shows a hexagonal structure typical of phyllosilicates in (*hk*0) reflection.

Supplementary Table 1 | Whole-rock geochemical data of major elements and carbon.

Formation	Lithology	Sample ID	Depth m	SiO_2 %	Al_2O_3 %	Fe_2O_3 %	MnO %	MgO %	CaO %	Na_2O %	K_2O %	TiO_2 %	P_2O_5 %	S_{tot} wt%	C_{tot} wt%	C_{org}* wt%
FB2b	Pyritized	FPS_s5-mat	1,3	23.2	10.1	38.4	0.1	1.35	0.59	0.19	1.88	0.14	BDL	29.37	0.69	0.56
	MRS	DB_s4-mat	2,9	32.87	9.71	29.74	0.16	1.63	1.27	0.17	1.81	0.18	BDL	22.02	3.12	2.85
		FPS_s4-mat	3,7	21.23	8.26	41.49	0.09	1.81	0.81	0.14	1.34	0.12	BDL	24.81	0.77	0.59
		FPS_s3-mat	3,8	26.69	9.62	35.26	0.12	1.87	0.86	0.19	1.62	0.15	BDL	28.45	1.36	1.18
		DB_s3-mat	4	24.17	10.5	29.86	0.08	1.44	0.85	0.19	1.97	0.15	BDL	26.9	0.53	0.35
		DB_s2-mat	4	27.84	11.83	20.82	0.07	1.44	0.51	0.21	2.28	0.2	BDL	23.88	X	X
		FR_s1-mat	4	51.3	15.74	10.33	0.24	2.43	2.08	0.31	3.04	0.34	BDL	6.46	3.52	3.08
	Black	WS_s1-sed	0,3	58.93	16.69	3.69	0.49	3.43	2.26	0.34	2.95	0.33	BDL	0.09	2.2	1.72
	shales	KS_s1-sed	1	61.85	15.14	3.81	0.23	3.02	2.07	0.3	2.83	0.42	0.2	0.09	2.91	2.47
		FPS_s5-sed	1,3	56.35	16.72	2.39	0.47	3.03	2.65	0.34	3.16	0.39	0.16	0.22	4.98	4.41
		AFBSO-12	1,4	62.88	18.65	3.44	0.07	1.96	0.37	0.39	3.63	0.41	0.17	X	X	X
		MLS_s7-sed	1,9	61.55	13.22	6.99	0.22	4.77	2.24	0.22	1.57	0.37	BDL	0.32	2.11	1.63
		AFBSO-9	2,5	54.74	12.06	6.5	0.74	5.55	5.21	0.23	1.47	0.32	0.12	X	3.8	2.68
		DB_s4-sed	2,9	58.09	17.07	2.81	0.44	3.06	2.67	0.33	3.32	0.39	0.13	0.13	3.83	3.26
		AFBSO-7	3,5	77.68	13.28	0.35	BDL	0.53	BDL	0.21	2.84	0.29	BDL	X	2.47	2.47
		FPS_s4-sed	3,7	57.81	18.6	2.2	0.26	2.74	2.07	0.35	3.73	0.39	0.16	0.16	2.62	2.18
		FPS_s3-sed	3,8	57.66	16.08	2.34	0.31	2.99	2.59	0.36	2.95	0.38	0.16	0.17	5.04	4.48
		AFBSO-6	3,9	66.18	12.04	8.87	0.11	3.09	0.24	0.22	1.35	0.26	0.15	X	3.64	3.59
		DB_s2-sed	4	56.11	17.85	2.15	0.3	2.87	2.24	0.36	3.59	0.39	BDL	0.16	4.59	4.11
		FR_s1-sed	4	58.3	18.86	2.36	0.24	2.86	2.18	0.38	3.79	0.4	0.14	0.2	2.73	2.26
		MLS_s8-sed	4,5	54.3	20.73	3.87	0.48	3.1	2.1	0.39	4.3	0.44	BDL	1.02	1.86	1.41
		MLS_s9-sed	4,5	56.29	19.14	3.02	0.37	2.99	2.09	0.37	3.87	0.41	BDL	0.34	1.77	1.32
FB2a	Non-	MLS_s1-mat	5	33.74	22.58	13.1	0.32	4.13	1.01	0.36	4.17	2.77	0.56	4.16	5.84	5.63
	pyritized	LP_s3-mat	5	41.35	27.69	5.03	0.1	3.5	0.34	0.59	5.34	1.91	0.34	0.19	3.74	3.67
	MRS	MLS_s2-mat	5	33.09	23.3	9.45	0.1	4.12	0.43	0.28	4.04	3.39	0.36	3.08	9.69	9.6
		MLS_s3-mat	5	38.3	20.66	13.32	0.16	4.45	0.79	0.29	3.46	2.34	0.65	2.61	4.44	4.27
		MLS_s4-mat	5	35.62	23.78	5.69	0.11	4.35	0.68	0.28	4.44	3.21	0.51	0.36	10.59	10.44
		MLS_s5-mat	5	35.56	24.54	6.41	0.09	4.67	0.6	0.29	4.29	3.13	0.49	0.42	9	8.87
		EST_s3-mat	5	31.67	27.89	3.47	0.03	5.62	0.03	0.4	2.78	0.37	0.1	0.36	16.97	16.96
		EST_s4-mat	5	31.66	28.67	4.25	0.05	6.76	0.03	0.33	2.3	0.23	0.05	0.29	X	X
		MLS_s3-mat	9	44.31	31.77	2.48	0.03	2.9	0.16	0.49	5.74	0.87	0.12	0.84	1.6	1.57
		EST_s1-mat	13,2	36.27	31	2.72	0.01	5.57	0.1	0.5	3.56	0.36	0.02	0.17	11.09	11.07
		EST_s2-mat	13,2	38.54	31.4	2.48	0.02	4.03	0.2	0.63	5.01	0.33	0.17	0.15	X	X
	Sandstones	MLS_s1-sed	5	89.24	4.66	0.97	0.11	0.96	0.65	0.1	0.6	0.03	BDL	X	0.45	0.31
		LP_s3-sed	5	93.65	2.46	0.66	0.07	0.63	0.6	0.04	0.32	BDL	BDL	X	0.52	0.39
		MLS_s2-sed	5	89.25	3.21	0.79	0.27	1.17	1.22	0.16	0.27	0.03	BDL	X	0.83	0.57
		AFBSO-3	5	96.83	0.95	0.08	0.01	0.08	BDL	*0.01*	0.08	0.02	BDL	X	0.36	0.36
		LP_s2-sed	6	95.03	2.08	0.56	0.01	0.39	0.06	0.04	0.19	0.02	0.03	X	0.31	0.3
		MLS_s3-sed	9	94.48	2.85	0.68	0.03	0.58	BDL	0.03	0.21	0.04	BDL	X	0.42	0.42
		EST_s1-sed	13,2	94.74	1.99	0.57	BDL	0.2	BDL	*0.01*	0.28	BDL	BDL	0.02	0.58	0.58
		EST_s2-sed	13,2	95.98	1.47	0.63	BDL	0.17	0.07	0.02	0.18	0.02	0.07	X	X	X

FPS: flat pyritized mat; LP: linear pattern; DB: domal buildup; WS: wrinkle structure; KS: "kinneyia" structure; MLS: mat-layer structure; FR: "fairy-ring" structure; EST: "elephant-skin" texture[11].

BLD: below detection limit. Italicized numbers reflect samples where half or more of the analyses were below detection limits for that element.

Organic carbon is calculated as follow: $C_{org} \approx C_{tot} - (12*CaO/56)$; $C_{org} = C_{tot}$ when CaO contents < BLD.

X: Not analysed

Supplementary Table 2 | Pairwise comparisons between all pairs of groups.

	Pyritized MRS (FB2b)	Black shales (FB2b)	Non-pyritized MRS (FB2a)	Sandstones (FB2a)
Pyritized MRS (FB2b)	-			
Black shales (FB2b)	**0.008**	-		
Non-pyritized MRS (FB2a)	**$8.0*10^{-05}$**	**$4.9*10^{-10}$**	-	
Sandstones (FB2a)	**$3.0*10^{-08}$**	**$2.9*10^{-06}$**	**$1.0*10^{-14}$**	-

p-value are given. Bold p-value (< 0.05) are significant.

Supplementary Table 3 | Structural parameters from the simulation of experimental XRD patterns of Ca-saturated samples after air-dried treatment and glycolation.

Samples	Chlorite		Mica		Illite		Random MLM				Ordered MLM			
	Rel. Ab. (%)	N	Rel. Ab. (%)	N	Rel. Ab. (%)	N	Rel. Ab. (%)	N	Layer Prop. (%) I/S	R	Rel. Ab. (%)	N	Layer Prop. (%) I/S	R
MRS	22	14-40	40	10-25	15	4-19 (AD)<br>5-12 (EG)	-	-	-	-	23	10-25 (AD)<br>4-19 (EG)	92/8<br>90/10	3
					**22**		**-**	**-**	**-**	**-**	**34**			
Sediment	13	12-25	31	22-38	22	7-11 (AD)<br>5-19 (EG)	13	4-10	35/65	0	21	11-20 (AD)<br>11-16 (EG)	91/9 (AD)<br>88/12 (EG)	3

Rel. Ab., relative abundance; N, number of layers in coherent scattering domain with a lognormal distribution; Layer Prop., layer proportion in the I-S MLM; R, Reichweite parameter; AD, air dry; EG; ethylene-glycol.

We cannot compare the initial results at constant volume due to changes in the reactivity of diagenetic clay fraction, which is indicated by the concentrated detrital fraction (mica + chlorite, 62%) in the MRS relative to the sediments (44%). Therefore, bold relative abundances correspond to the calculated diagenetic fraction where the proportions of insoluble detrital components in the MRS are considered unchanged relative to the sediments (*i.e.*, 44%). The transformation from R0 to R3 I-S MLM as well as intense dissolution in the MRS represent the reactive clay fraction.

Supplementary references

1. Taylor, S. R. & McLennan, S. M. Chemical Composition and Element Distribution in the Earth's Crust. in *Encyclopedia of Physical Science and Technology* (ed. Roberts, A. M.) 697–719 (Academic Press, 2001).
2. Środoń, J. & Eberl, D. D. Illite. in *Review in Mineralogy 13, Micas* (ed. Bailey, S. W.) 495–544 (Mineralogical Society of America, 1984).
3. Horie, K., Hidaka, H. & Gauthier-Lafaye, F. U-Pb geochronology and geochemistry of zircon from the Franceville series at Bidoudouma, Gabon. *The 15th Annual Goldschmidt Conference* (2005).
4. Bros, R., Stille, P., Gauthier-Lafaye, F., Weber, F. & Clauer, N. Sm-Nd isotopic dating of Proterozoic clay material: an example from the Francevillian sedimentary series, Gabon. *Earth Planet. Sci. Lett.* **113**, 207–218 (1992).
5. Gancarz, A. J. U-Pb age (2.05×10^9 years) of the Oklo uranium deposit. *The Natural Fission Reactors: Annual International Atomic Energy Agency Conference* (1978).
6. Mouélé, I. M. *et al.* 2.9–1.9 Ga paleoalterations of Archean granitic basement of the Franceville basin (Gabon). *J. Afr. Earth Sci.* **97**, 244–260 (2014).
7. Gauthier-Lafaye, F. & Weber, F. Natural nuclear fission reactors: time constraints for occurrence, and their relation to uranium and manganese deposits and to the evolution of the atmosphere. *Precambrian Res.* **120**, 81–100 (2003).
8. El Albani, A. *et al.* Large colonial organisms with coordinated growth in oxygenated environments 2.1 Gyr ago. *Nature* **466**, 100–104 (2010).
9. El Albani, A. *et al.* The 2.1 Ga Old Francevillian Biota: Biogenicity, Taphonomy and Biodiversity. *PLoS ONE* **9**, e99438 (2014).
10. Billault, V., Beaufort, D., Patrier, P. & Petit, S. Crystal chemistry of Fe-sudoites from uranium deposits in the Athabasca basin (Saskatchwan, Canada). *Clays Clay Miner.* **50**, 70–81 (2002).
11. Aubineau, J. *et al.* Unusual microbial mat-related structural diversity 2.1 billion years ago and implications for the Francevillian biota. *Geobiology* **16**, 476–497 (2018).
